# Are temporal patterns of sitting associated with obesity among blue-collar workers? A cross sectional study using accelerometers

**DOI:** 10.1186/s12889-016-2803-9

**Published:** 2016-02-13

**Authors:** Nidhi Gupta, David M. Hallman, Svend Erik Mathiassen, Mette Aadahl, Marie Birk Jørgensen, Andreas Holtermann

**Affiliations:** National Research Centre for the Working Environment, Copenhagen, Lerso Parkalle 105, 2100 Copenhagen, Denmark; Centre for Musculoskeletal Research, Department of Occupational and Public Health Sciences, University of Gävle, SE-80176 Gävle, Sweden; Research Centre for Prevention and Health, The Capital Region of Denmark, Glostrup Hospital, 2600 Glostrup, Denmark; Department of Public Health, Faculty of Health and Medical Sciences, University of Copenhagen, 1014 Copenhagen, Denmark; Institute of Sports Science and Clinical Biomechanics, University of Southern Denmark, 5230 Odense, Denmark

**Keywords:** Brief sitting bouts, prolonged sitting bouts, Total sitting time, Sedentary behavior, Prolong sitting, Interrupted sitting, Working day, Physical activity

## Abstract

**Background:**

Little is known about associations of *temporal* patterns of sitting (i.e., distribution of sitting across time) with obesity. We aimed investigating the association between temporal patterns of sitting (long, moderate and brief uninterrupted bouts) and obesity indicators (body mass index (BMI), waist circumference and fat percentage), independently from moderate-vigorous physical activity (MVPA) and total sitting time among blue-collar workers.

**Methods:**

Workers (*n* = 205) wore Actigraph GT3X+ accelerometers on the thigh and trunk for 1–4 working days. Using the validated Acti4 software, the total sitting time and time spent sitting in *brief* (≤5 mins), *moderate* (>5 and ≤30 mins), and *long* (>30mins) bouts on working days were determined for the whole day, and for leisure and work separately. BMI (kg/m^2^), waist circumference (cm) and fat percentage were objectively measured.

**Results:**

Results of linear regression analysis adjusted for multiple confounders indicated that brief bouts of sitting was negatively associated with obesity for the whole day (BMI, *P* < 0.01; fat percentage, *P* < 0.01; waist circumference, *P* < 0.01) and work (BMI, *P* < 0.01; fat percentage, *P* < 0.01; waist circumference, *P* < 0.01), but not for leisure. Sitting time in long bouts was positively associated with obesity indicators for the whole day (waist circumference, *P* = 0.05) and work (waist circumference, *P* = 0.01; BMI, *P* = 0.04), but not leisure.

**Conclusions:**

For the whole day as well as for work, brief bouts and long bouts of sitting showed opposite associations with obesity even after adjusting for MVPA and total sitting time, while sitting during leisure did not show these associations. Thus, the temporal distribution of sitting seems to influence the relationship between sitting and obesity.

## Background

Based on recent estimates, 30–70 % of the European adults are obese [1] with high obesity rates particularly among workers in lower socioeconomic groups such as blue-collar workers [2, 3]. Obesity is associated with an increased risk for all-cause mortality [4], metabolic syndrome, diabetes, cardiovascular diseases, and cancer [5, 6].

Sedentary behavior has increased in western countries where many adults spend 70 % or more of their waking hours in sitting [7]. Extensive sitting is also prevalent among working populations [8]. According to recent studies, blue-collar workers such as assemblers and drivers spent as much as 50 % of the working hours being sedentary [9, 10]. Moreover, due to the nature of work tasks (e.g. long transportation, assembly line work or surveillance), blue collar workers may sit for longer period of time or due to organizational reasons (e.g., low decision latitude), these workers may have limited autonomy over deciding on breaking up the prolonged periods. Among these workers, substantial leisure time sitting has also been observed [9, 11]. Accordingly, these workers also have a higher prevalence of all-cause mortality and chronic diseases such as ischemic heart diseases compared to white-collar workers [12, 13].

Studies have found a positive association between both self-reported and objectively measured total sitting time and obesity indicators [14], even when adjusted for moderate to vigorous physical activity levels (MVPA) [15–17]. However, few studies have addressed the importance of objectively measured temporal patterns of sitting (i.e. how sitting is distributed across time) with respect to obesity indicators [18, 19]. Recent studies have found that a larger occurrence of objectively measured long bouts of sitting (i.e., uninterrupted sitting bouts >30 min) is associated with negative health consequences such as increased cardio-metabolic risk [20, 21]. Sitting in brief bouts is not considered a health hazard to the same extent [18–20]. A further issue is that those few studies investigating the association between temporal patterns of sitting and health outcomes have primarily measured sitting time using accelerometer counts [18, 20, 22, 23]. Count-based accelerometer thresholds to determine sitting are criticized due to their inability to accurately differentiate sitting from standing postures, thus giving incorrect information about temporal patterns of sitting [24].

Additionally, those studies have almost exclusively focused on whole day and/or leisure time [21, 25], not discriminating between the effects of sitting at work and during leisure. Work and leisure periods differ in sitting patterns [26, 27] and it is important to understand the contribution of each of these domains to the overall risk of being obese.

We aimed to investigate the extent to which objectively measured temporal patterns of sitting are associated with body mass index (BMI), waist circumference, and fat percentage, accounting for total sitting time and objectively measured MVPA time, among blue-collar workers. We hypothesized that time spent in long sitting bouts during the whole day, as well as during work and leisure time separately is positively associated with obesity indicators, while time spent in brief sitting bouts is negatively associated with those indicators, after accounting for total sitting time and MVPA.

## Methods

### Design and study population

The current cross-sectional sample consisted of blue-collar workers from ‘The New method for Objective Measurements of physical Activity in Daily living (NOMAD)’ study. The design, methods, and inclusion and exclusion criteria are described in detail elsewhere [9, 10, 28].

### Ethics and consent

The study was approved by the Ethics Committee for the Capital Region of Denmark (journal number H-2-2011-047) and conducted in accordance with the Helsinki declaration. Informed consent was obtained from all individual participants included in the study.

### Availability of data and materials

The datasets supporting the conclusions of this study are available upon request from Andreas Holtermann, National Research Centre for the Working Environment, Copenhagen, Denmark (aho@nrcwe.dk).

### Measurements

Data were collected between August 2011 and April 2012. Workers interested in participating in the study underwent anthropometric and body composition measurements and completed a short questionnaire. Also diurnal objective measurements of sedentary behavior and physical activities were initiated by equipping the workers with two accelerometers (Actigraph GT3X+, Pensacola, FL, USA) placed at the standardized position directly on the skin of the thigh and trunk [9, 29]. Additionally, workers were given a paper diary for noting start and end of work, bedtime in the evening, and wake-up in the morning [9]. On day four, workers returned the measurement equipment.

#### Objectively measured BMI, fat percentage and waist circumference

The weight and fat percentage (TANITA model BC418 MA, TANITA corporation, Tokyo, Japan), height (Seca model 123, Birmingham, UK) and waist circumference (Seca model 201, Birmingham, UK) of the workers were objectively measured. Their BMI (kg/m^2^) was calculated as weight (kg) divided by height (m) squared.

#### Objectively measured sitting time and time spent in moderate-vigorous physical activity

The objectively measured sitting time and moderate-vigorous physical activity (MVPA) were determined using a custom-made MATLAB program, Acti4 estimating the time-line of periods of physical activities and body postures (type, duration, and intensity) across the day(s) with a sensitivity and specificity of more than 98 % and 99 %, respectively [29]. Using the individual’s reference measurement values of the thigh and trunk accelerometer, the occurrence of sitting postures were identified according to the procedure described by Gupta et al. [9]. Sitting was identified when the inclination of the thigh accelerometer was above 45° and that of the trunk accelerometer below 45° [29]. Acti4 has previously been shown to determine sitting posture during free living conditions with a sensitivity of 98 % and a specificity of 93 % [29]. The occurrences of fast walking, running, stair climbing, and cycling were identified based on procedures explained in previous studies [29, 30].

Only workers with at least one valid, measured working day were included in the analyses. Previous studies have suggested that at least one valid day of measurement is necessary for estimating physical activities [31–34]. A working day was considered valid if it comprised at least 10 h of wear time and included at least 4 h of self-reported work [9, 10]. For the specific analyses of work and leisure, workers were included if their recordings offered at least one day with a valid period of work or leisure data, respectively. A work period was considered valid if it comprised at least 75 % of the individual’s average reported working time per day. A leisure period was considered valid if it comprised at least 4 h of leisure time, which needed to correspond to at least 75 % of the individual’s average reported leisure time per day. Work was identified from the self-reported information in the diary about hours spent at the primary occupation, and leisure was defined as the remaining waking hours. These criteria were chosen so as to prevent bias due to inclusion of very unrepresentative data in the analyses, and to reflect optimal daily wear time for valid measurements of sitting time [9].

Subsequently, based on the measured timeline of the sitting on working days, total sitting time retrieved in the following domains were divided by the number of valid measured days; (a) *whole day* (b) *work* and (c) *leisure*. Additionally, the MVPA of each worker was calculated by adding the total time spent in fast walking, running, cycling, and stair climbing divided by number of days measured.

#### Exposure variation analysis (EVA) of sitting time

The temporal patterns of sitting were determined using EVA [35], modified to address sedentary behavior. Thus, EVA was utilized to categorize uninterrupted sitting time into ‘long bouts’ (LB; i.e. average time/day spent in uninterrupted sitting bouts >30 min), ‘moderate bouts’ (MB; i.e. average time/day spent in uninterrupted sitting bouts >5 and ≤30min), and ‘brief bursts’ (BB; i.e. average time/day spent in uninterrupted sitting bouts ≤5mins) [21, 36].

#### Potential confounders

Potential confounders were selected *a priori* based on previous studies on risk factors of obesity [37]. Age, gender, influence at work, and smoking behavior were determined according to previous studies [9, 10, 28] while the MVPA and total sitting time were measured objectively (as explained above). Poor dietary habits were determined using following single item ‘*How often do you usually eat and/or drink candy, ice cream, chocolate, soft drinks*’ with four responses (daily, 3–4/week, 1–2/week, and rarely). Alcohol intake was determined by following item ‘*On average, how much alcohol do you drink during the working days and on non-working days* with responses in number of units per day.

### Statistical analysis

All statistical analyses described below were performed for each of the three time domains, i.e., whole day, work and leisure; and for each of the three obesity indicators, i.e., BMI, fat percentage and waist circumference.

The unadjusted association between total sitting time, as the independent variable, and each obesity indicator as the dependent variable was determined using ordinary linear least-square regression analysis. This analysis was then adjusted in two steps; i.e. model 1: for age and gender; and model 2 for the variables in model 1 and influence at work, smoking behavior, MVPA, dietary habits, alcohol intake, and total measured time in the domain under study.

Similar linear regression models were resolved to determine associations between each EVA derivative [LB, MB, and BB of sitting] and the obesity indicator, with an additional model adjusting for total sitting time in the domain under study (Model 3). Specifically for the work and leisure domains, even a fourth model was applied, adjusting for sitting in the complementary domain (Model 4) to determine the independent effect of sitting in the modelled domain.

The assumptions of linearity, and residuals being normally distributed and homoscedastic were fulfilled for all regression models. Additionally, no major multi-collinearity issues were detected (tolerance index >0.20 VIF values <5 [38]) for the independent variables.

## Results

**Fig. 1:**
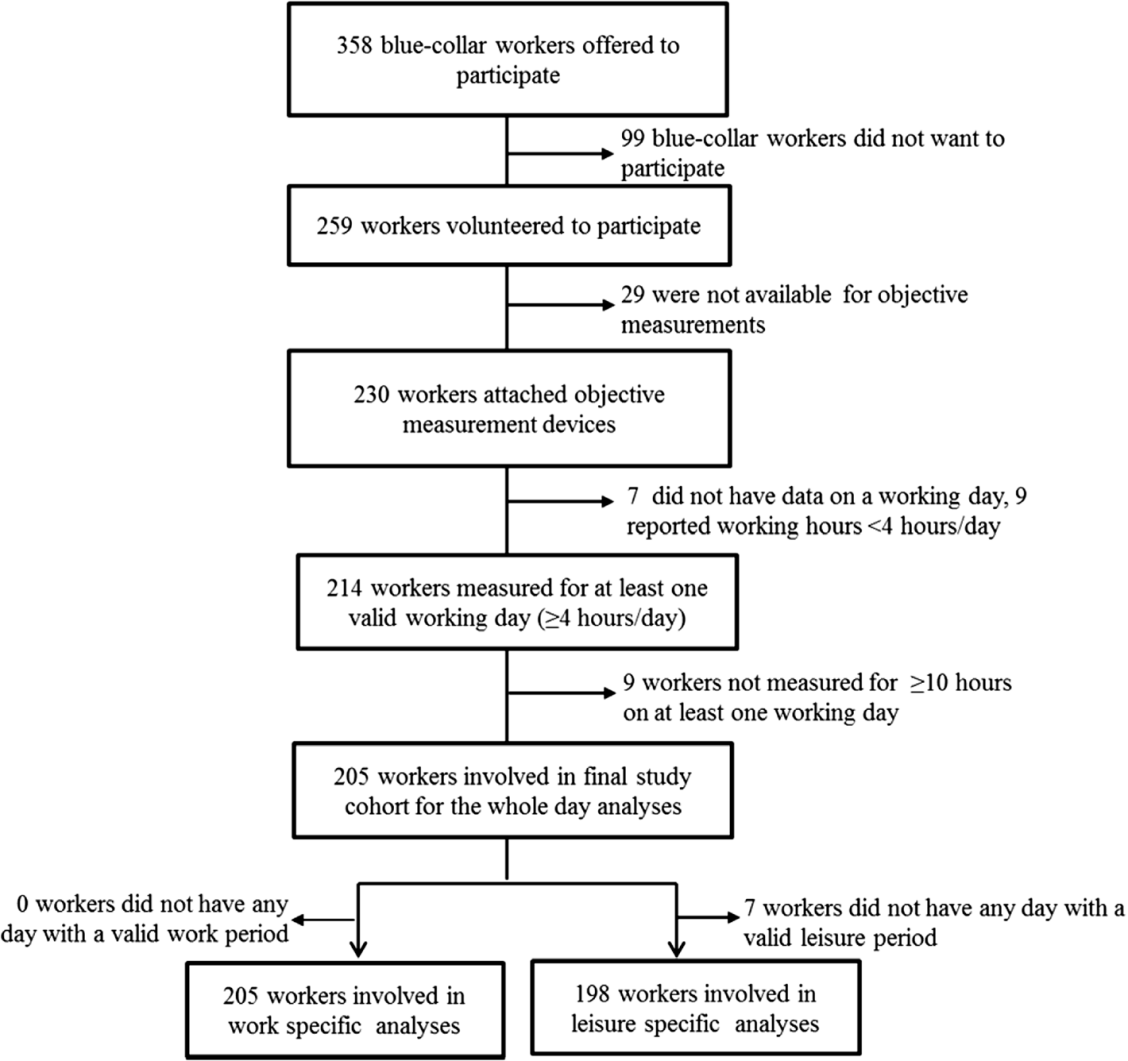
Recruitment process of the study group in Denmark

The recruitment process is shown in Fig. 1 and the descriptives of the workers are shown in Table 1.

**Table 1:** Characteristics of the Danish blue-collar workers included in the statistical analysis

Variables	Number	Percent	Mean	SD
Age (years)	205		44.8	9.7
Gender				
Males	120	59		
Females	85	41		
Influence at work in 0–100 %	203		44.0	23.5
Alcohol (number of units/week)^a^	201		1.5	2.5
Smokers	84	43		
Poor dietary habits [1(daily)-4(rarely)]	202		2.6	0.9
Daily	28	14		
3–4 times/week	54	27		
1–2 times/week	85	42		
Rarely	35	17		
BMI (kg/m^2^)	205		26.4	5.0
Fat percentage	201		25.4	9.9
Waist circumference (cm)	205		92.5	13.6
MVPA (h/day)	205		2.0	0.8
Total measured time (h/day)	205		16.7	1.5
Valid days of measurements	205		2.5	1.1
Total sitting time in whole day (h/day)	205		8.4	2.4
LB in whole day (h/day)	205		3.2	1.8
MB in whole day (h/day)	205		4.1	1.2
BB in whole day (h/day)	205		1.2	0.6
Total sitting time during work (h/day)	205		3.1	1.5
LB during work (h/day)	205		0.6	0.8
MB during work (h/day)	205		1.9	0.9
BB during work (h/day)	205		0.7	0.4
Total sitting time during leisure (h/day)	198		5.8	1.9
LB during leisure (h/day)	198		2.9	1.6
MB during leisure (h/day)	198		2.4	0.9
BB during leisure (h/day)	198		0.6	0.3

*BMI* body mass index, *MVPA* moderate-vigorous physical activity, *M* mean, *SD* standard deviation, *M* mean

^a^the alcohol intake is the average of number of units consumed per day on working and non-working days; LB = long sitting bouts (>30 mins), MB = moderate sitting bouts (>5 and ≤30 mins), BB = brief sitting bouts (≤5 mins)

The whole-day analyses included a total of 9,000 waking hours of accelerometer data, distributed among 507 valid days. On average, workers were measured for 16.7 (SD between workers 1.5) hours per day. About 80 % of the workers wore accelerometers for 2 valid days or more. In the specific analyses of work and leisure, a total of 4019 valid work hours and 3569 valid leisure hours were included.

On average, workers were sitting for more than 50 % of the total waking hours. Total sitting time was higher during leisure than work (Table 1). On average, workers spent most of their time in LB (i.e., >30 min), and least time in BB (i.e., 0–5 min) in all three domains. They spent more time in LB and MB (>5 and ≤30 min) during leisure than at work (Table 1). No marked difference between work and leisure domains was found for BB.

### Association of sitting time variables with obesity indicators

**Fig. 2:**
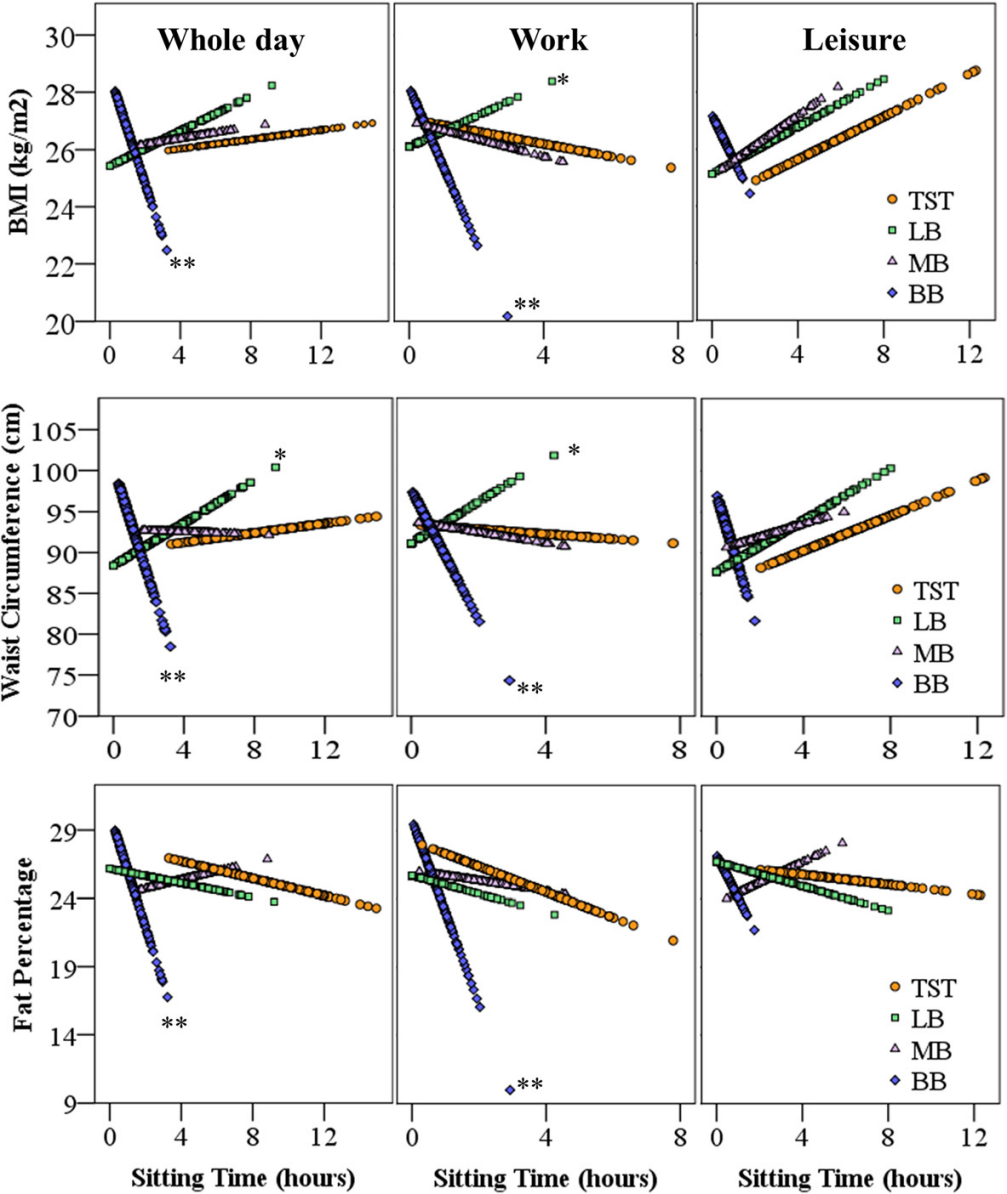
Regression plots illustrating unadjusted association between sitting variables^±^ and predicted obesity indicators^҂^ for all domains^ǂ^ BMI = body mass index, TST = total sitting time, LB = long sitting bouts (>30 mins), MB = moderate sitting bouts (>5 and ≤30 mins), BB = brief sitting bouts (≤5 mins). * and ** indicate p-values less than 0.05 and 0.01, respectively, for the regression coefficient being different from zero in the fully adjusted model. LB, MB, and BB. ^҂^BMI, waist circumference and fat percentage. ^ǂ^whole day, work, and leisure

Table 2 reports associations between sitting time variables (total sitting time, LB, MB, and BB) and obesity indicators (BMI, fat percentage and waist circumference). According to model 1, total sitting time was not significantly associated with obesity indicators, neither for the whole day nor for the work domain, as illustrated even in Fig. 2. Regression coefficients and their significance did not change markedly with further adjustment for confounders in model 2 and model 3. However, during leisure, after adjusting for confounders in models 2 and 3, we observed a tendency (*P* = 0.07–0.10) of a positive association between total sitting time and all obesity indicators.

**Table 2:** Standardized regression coefficients, measuring associations between sitting variables^±^ and obesity indicators^҂^ during all domains^ǂ^

Variable	Model	Whole day (*n* = 205)				Work (*n* = 205)				Leisure (*n* = 198)			
		B	Low 95%CI	High 95 % CI	P	B	Low 95%CI	High 95 % CI	P	B	Low 95 % CI	High 95 % CI	P
**BMI**													
Total sitting time	1	0.05	−0.24	0.34	0.74	−0.16	−0.64	0.31	0.50	0.30	−0.07	0.67	0.11
	2	0.23	−0.22	0.67	0.32	−0.03	−0.66	0.60	0.92	0.63	−0.14	1.40	0.11
	3					−0.02	−0.65	0.62	0.95	**0.65**	**−0.13**	**1.43**	**0.10**
LB (>30min)	1	0.22	−0.17	0.61	0.27	0.48	−0.43	1.39	0.30	0.32	−0.13	0.77	0.16
	2	0.44	−0.11	0.99	0.12	**0.96**	**−0.14**	**2.06**	**0.09**	0.29	−0.34	0.91	0.37
	3	0.41	−0.26	1.09	0.23	**1.33**	**0.05**	**2.62**	**0.04**	−0.15	−1.03	0.73	0.74
	4					**1.32**	**0.04**	**2.61**	**0.04**	−0.12	−1.00	0.75	0.78
MB (>5 and ≤30 mins)	1	0.11	−0.44	0.67	0.68	−0.18	−0.94	0.58	0.64	0.42	−0.33	1.17	0.27
	2	0.21	−0.42	0.85	0.51	−0.10	−1.04	0.85	0.84	0.44	−0.51	1.39	0.36
	3	0.04	−0.74	0.82	0.92	−0.15	−1.69	1.38	0.84	0.30	−0.66	1.27	0.53
	4					−0.17	−1.71	1.37	0.83	0.30	−0.67	1.27	0.55
BB (≤5 mins)	1	**−1.72**	**−2.89**	**−0.55**	**<0.01**	**−2.59**	**−4.17**	**−1.01**	**<0.01**	−1.15	−3.56	1.27	0.35
	2	**−1.59**	**−2.96**	**−0.21**	**0.02**	**−2.37**	**−4.11**	**−0.62**	**0.01**	−1.53	−4.58	1.52	0.32
	3	**−2.31**	**−3.81**	**−0.80**	**<0.01**	**−3.17**	**−5.21**	**−1.14**	**<0.01**	−1.27	−4.33	1.79	0.41
	4					**−3.06**	**−5.11**	**−1.01**	**<0.01**	−0.90	−3.94	2.14	0.56
**Waist circumference**													
Total sitting time	1	0.00	−0.71	0.72	0.99	−0.22	−1.41	0.97	0.71	0.46	−0.48	1.39	0.34
	2	0.05	−1.08	1.18	0.93	−0.10	−1.69	1.49	0.90	**1.80**	**−0.14**	**3.73**	**0.07**
	3					−0.05	−1.64	1.54	0.95	**1.79**	**−0.17**	**3.76**	**0.07**
LB (>30min)	1	0.71	−0.27	1.68	0.15	1.89	−0.38	4.16	0.10	0.84	−0.28	1.97	0.14
	2	**1.19**	**−0.20**	**2.58**	**0.09**	**3.22**	**0.46**	**5.98**	**0.02**	1.19	−0.39	2.76	0.14
	3	**1.71**	**0.01**	**3.40**	**0.05**	**4.48**	**1.28**	**7.67**	**0.01**	0.32	−1.91	2.54	0.78
	4					**4.39**	**1.20**	**7.59**	**0.01**	0.39	−1.81	2.59	0.73
MB (>5 and ≤30 mins)	1	−0.02	−1.40	1.36	0.98	−0.07	−1.97	1.83	0.94	0.20	−1.68	2.08	0.83
	2	−0.07	−1.68	1.53	0.93	−0.48	−2.85	1.90	0.69	0.71	−1.69	3.11	0.56
	3	−0.17	−2.14	1.80	0.86	−0.94	−4.80	2.92	0.63	0.30	−2.13	2.73	0.81
	4					−0.96	−4.83	2.91	0.62	0.32	−2.13	2.76	0.80
BB (≤5 mins)	1	**−6.42**	**−9.27**	**−3.56**	**<0.01**	**−8.23**	**−12.12**	**−4.33**	**<0.01**	**−7.23**	**−13.20**	**−1.27**	**0.02**
	2	**−6.64**	**−10.02**	**−3.26**	**<0.01**	**−7.37**	**−11.71**	**−3.02**	**<0.01**	**−7.56**	**−15.19**	**0.08**	**0.05**
	3	**−8.19**	**−11.90**	**−4.49**	**<0.01**	**−9.87**	**−14.91**	**−4.83**	**<0.01**	**−6.85**	**−14.49**	**0.80**	**0.08**
	4					**−9.33**	**−14.38**	**−4.27**	**<0.01**	−5.72	−13.26	1.82	0.14
**Fat percentage**													
Total sitting time	1	−0.08	−0.49	0.34	0.72	−0.54	−1.22	0.14	0.12	0.27	−0.27	0.82	0.32
	2	0.27	−0.38	0.91	0.41	−0.37	−1.28	0.54	0.42	**0.96**	**−0.14**	**2.07**	**0.09**
	3					−0.35	−1.26	0.56	0.45	**0.93**	**−0.19**	**2.05**	**0.10**
LB (>30min)	1	0.11	−0.46	0.68	0.71	0.10	−1.23	1.42	0.88	0.15	−0.52	0.82	0.67
	2	0.42	−0.38	1.22	0.30	0.42	−1.18	2.02	0.61	0.19	−0.72	1.10	0.68
	3	0.34	−0.64	1.32	0.49	1.05	−0.83	2.93	0.27	−0.72	−1.99	0.54	0.26
	4					1.04	−0.84	2.93	0.28	−0.70	−1.96	0.56	0.28
MB (>5 and ≤30 mins)	1	0.15	−0.65	0.96	0.71	−0.52	−1.63	0.59	0.35	**0.90**	**−0.16**	**1.97**	**0.09**
	2	0.58	−0.34	1.50	0.21	−0.03	−1.41	1.35	0.97	**1.30**	**−0.05**	**2.65**	**0.06**
	3	0.54	−0.59	1.66	0.35	1.09	−1.15	3.32	0.34	1.10	−0.27	2.48	0.12
	4					1.09	−1.14	3.32	0.34	1.11	−0.27	2.50	0.11
BB (≤5 mins)	1	**−2.92**	**−4.57**	**−1.26**	**<0.01**	**−4.20**	**−6.44**	**−1.95**	**<0.01**	−2.40	−5.83	1.03	0.17
	2	**−2.73**	**−4.68**	**−0.78**	**0.01**	**−3.88**	**−6.36**	**−1.39**	**<0.01**	−2.78	−7.13	1.57	0.21
	3	**−3.78**	**−5.91**	**−1.65**	**<0.01**	**−4.57**	**−7.47**	**−1.66**	**<0.01**	−2.40	−6.75	1.96	0.28
	4					**−4.30**	**−7.22**	**−1.38**	**<0.01**	−1.77	−6.07	2.52	0.42

Model 1. Adjusted for age and gender. Model 2. Adjusted for variables in model1 and smoking status, alcohol intake, diet habits, influence at work, MVPA and total measured time during the corresponding domain. Model 3. Adjusted for the variables in model 2 and total sitting time in the domain under study. Model 4. Adjusted for the variables in model 3 and the EVA variable under study in the opposite domain

*BMI* body mass index, *LB* long sitting bouts (>30 mins), *MB* moderate sitting bouts (>5 and ≤30 mins), *BB* brief sitting bouts (≤5 mins). Coefficients are given with 95 % confidence interval (CI), and p-value pertaining to the null-hypothesis of zero effect, significant effects (*p* < 0.05) are shown in boldface. ^±^total sitting time, LB, MB, and BB. ^҂^BMI, waist circumference and fat percentage. ^ǂ^whole day, work, and leisure

Sitting time spent in BB was negatively associated with all obesity indicators in model 1, both during the whole day and at work (Table 2). The associations persisted after further adjustments in model 2, 3 and 4. During leisure, models 1 and 2 indicated a negative association between BB and waist circumference, which persisted in models 3 and 4, although it was no longer statistically significant.

A tendency for a positive association of LB at work with waist circumference and BMI was found in model 1, and it became stronger and more significant with further adjustments in model 2, 3 and 4. For the whole day, LB was positively associated with waist circumference only. No clear association was found between LB during leisure and any obesity indicator. LB and fat percentage were not associated in any time domain.

No associations were found between sitting time spent in MB and any obesity indicators in any of the time domains.

## Discussion

This study showed that sitting in BB throughout the day and at work was negatively associated with BMI, fat percentage and waist circumference, while sitting in LB was positively associated with waist circumference and BMI, but not with fat percentage. Temporal patterns of sitting during leisure time were not significantly associated with any obesity indicator, while total time sitting during leisure showed a tendency of being positively associated with the obesity indicators.

As hypothesized, we found a negative association between BB of sitting and obesity indicators during whole day and work. The time spent in BB at work in this population was, on average, 42 min (SD 24 min). According to Fig. 2, spending 10 min in BB (0–5 min) at work was associated with waist circumference of ~97 cm while spending 40 min in BB was associated with waist circumference of ~92 cm. The associations with BB of sitting persisted after adjusting for several potential confounders, including MVPA and total sitting time. This finding suggests that those who spent more time in BB of sitting were less likely to be obese than those who spent less time in BB, independent of their total sitting time and level of MVPA. This result agrees with some previous studies on sedentary behavior such as breaks in prolonged sitting (e.g., transitions from a sedentary to an active state lasting ≥1min) and obesity indicators, after adjusting for total sitting time and MVPA [18, 19]. Sitting in BB in our study could be considered as a ‘proxy’ for ‘breaking up’ sustained sitting periods sitting by various physical activities associated with blue-collar work.

Our hypothesis was also confirmed with respect to a positive association between sitting in LB and obesity indicators (i.e., BMI and waist circumference). In other words, workers who spent less time in LB of sitting were less obese and vice versa. For example, Fig. 2 shows that a worker spending 1 h at work in LB had waist circumference of ~94 cm while a worker spending 3 h in LB had waist circumference of ~99 cm. The effect of LB persisted after adjusting for potential confounders including MVPA and total sitting time. This suggests, together with the findings on BB, that the temporal distribution of sitting time is important to obesity in its own right, and that this relationship is independent of the extent of MVPA and total sitting time.

While based on animal studies, a possible and previously suggested explanation for the inverse associations of LB and BB with obesity indicators could be that prolonged sitting may lead to a loss of contractile stimulation of weight bearing muscles [39, 40]. This could suppress lipoprotein lipase activity which, in turn, could impair several aspects of lipid metabolism (such as triglycerides uptake and HDL production) [39, 40] and contribute to the development of obesity on the long term. On the other hand, frequent interruptions of sitting could facilitate lipid metabolism and glucose removal from the blood due to intermittent muscle contractions [41, 42], which may, in the long term, decrease the probability of becoming obese. Frequent interruptions of sitting by short breaks could also lead to larger total energy expenditure than fewer but longer breaks, and thus a more pronounced effect on obesity indicators, as suggested by others [18]. However, these hypotheses require further investigation, since we used a cross-sectional study design and did not measure any metabolic variables.

Most of our results concerning associations between temporal patterns of sitting and obesity indicators persisted after adjusting for total sitting time. Total sitting time *per se* was not significantly associated with any obesity indicators in the analyses of whole days and work, and tended to be in analyses of leisure time. These findings agree with previous studies reporting no significant associations between objectively measured total sitting time and obesity indicators such as BMI [43, 44], weight status [43, 44], percent body fat, waist hip ratio [45], and waist circumference [44]. The total sitting time is distributed in sitting periods of different durations, which may, according to our results, have different direction of association with obesity. Addressing only the total sitting time may therefore mask important associations of the structure of sitting time with obesity. Our results, suggesting that the temporal pattern of sitting is important to obesity, independent of the total sitting time, encourage interventions on the temporal pattern of sitting for preventing obesity.

Another interesting finding in our study was the lack of clear associations between the temporal pattern of sitting and obesity indicators during leisure. We found a slight tendency of a negative association between BB of sitting and waist circumference. However, it did not reach significance after adjustment for total sitting time and BB of sitting during work. On the other hand, we found a tendency of a positive association between total sitting time at leisure and obesity outcomes. It could be that total sitting time is so long during leisure (on average 5.8 h, or 65 % of total measured time) that the temporal pattern of sitting gets less important. We also found that LB of sitting during leisure was not significantly associated with obesity indicators, as opposed to LB during work. Until now, to the best of our knowledge, no previous study has investigated this association specifically during leisure. One explanation that LB of sitting during leisure showed a weaker association could be that sitting behavior during leisure is more heterogeneous and, to a larger extent, associated with confounding factors such as eating snacks during TV viewing. This increased uncertainty of the contents of LB in leisure-time sitting which would, for statistical reasons, lead to a weaker association with any outcome.

We found that waist circumference was more strongly associated with temporal pattern of sitting, followed by BMI and fat percentage. Waist circumference is a measure of central adiposity in the relatively small visceral adipose tissue compartment, which has been shown to be closely related to physiological disturbances caused by weight gain than the total mass of adipose tissues in the body [46]. Thus it is of note that we observed a stronger association of temporal sitting patterns with waist circumference than with body fat and BMI, suggesting that temporal patterns of sitting are, indeed, relevant to obesity related health outcomes.

### Methodological considerations, strength and limitations

A major strength of our study is the study population of blue-collar workers varying little in socioeconomic status but offering a great variation in sitting time, yet with a considerable average prevalence of sitting, i.e. slightly more than 50 % of the time. Also, sitting time was measured using two accelerometers which allowed us to separate standing and lying from sitting. We also used a validated software, Acti4, discriminating activities with an excellent sensitivity and specificity [29]. Additionally, we utilized exposure variation analysis (EVA) to determine the temporal pattern of sitting. EVA is a versatile generic approach for quantifying the level and frequency of activities, as demonstrated by the use of EVA for analyses of, e.g. working postures [47] and physical activity intensities [36].

In our analyses, we adjusted for potential confounders such as MVPA and total sitting time [17, 21, 25] to identify any independent association of temporal patterns of sitting with obesity indicators. However, adjusting for MVPA and total sitting time did not change the results to any major extent. Our results also persisted after adjustment for wear time, indicating no bias due to between-worker differences in measurement time. Moreover, we also mutually adjusted for sitting variables during work and leisure when investigating their independent association with obesity indicators.

The main limitation of the study is the cross-sectional study design, which does not allow inferences about causal relationships between sitting patterns and obesity. Thus, further prospective studies assessing the direction of the association between accurately measured temporal patterns of sitting at work and leisure and obesity are needed, as a basis for discussing causation. Since our study included a convenience sample of companies with a high fraction of blue-collar workers, our results may not be generalizable to the general population of blue-collar workers in Denmark, let alone in industrialized countries in general.

## Conclusion

Among blue-collar workers, a temporal distribution of sitting characterized by long uninterrupted bouts and few brief bouts during whole days and during work was found to be associated with increased waist circumference and BMI, even after adjustment for total sitting time and moderate-to-vigorous physical activity. Leisure time sitting did not show such associations. Our results suggest that the temporal pattern of sitting may be important to the risk of being obese, independently of the total sitting time, even though prospective studies are needed to confirm any causal relationships.

## Abbreviations

B: regression coefficient
BB: brief bouts
BMI: body mass index
CI: confidence interval
EVA: exposure variation analysis
LB: long bouts
M: mean
MB: moderate bouts
MVPA: moderate to vigorous physical activity
SD: standard deviation
TST: total sitting time
